# Studies on human body composition in Russia: past and present

**DOI:** 10.1186/s40101-022-00291-3

**Published:** 2022-05-03

**Authors:** Sergey G. Rudnev, Elena Z. Godina

**Affiliations:** 1 Marchuk Institute of Numerical Mathematics, Russian Academy of Sciences, Gubkin str. 8, Moscow, 119333 Russia; 2 Federal Research Institute for Health Organization and Informatics, Dobrolyubov str. 11, Moscow, 127254 Russia; 3 Anuchin Research Institute and Museum of Anthropology, Lomonosov Moscow State University, Mokhovaya str. 11, Moscow, 125009 Russia; 4 Russian State University of Physical Education, Sports, Youth and Tourism, Syrenevy Bulvar 4, Moscow, 105122 Russia

**Keywords:** Body composition, Anthropometry, BIA, DXA, Russia

## Abstract

Despite the presence of body composition studies in Russia, there are no current reviews on this topic, and the results are relatively rarely published abroad. Our aim was to describe the history and current state of this research work, to list unresolved problems, and to outline possible developmental trends. For completeness, in the initial part of the review, traditional research areas indirectly related to body composition studies are considered, namely, the analysis of biological variation of anthropometric parameters and somatotyping.

It can be seen that anthropometry and bioimpedance analysis (BIA) are mainly used to assess body composition in Russia. Other methods, such as double-energy X-ray absorptiometry (DXA), are utilized less often. The achievements include the common use of comprehensive anthropometry in anthropological studies, some advancements in clinical studies, approbation of potentially important methods such as the deuterium dilution method and three-dimensional laser-based photonic scanning, and ongoing mass population BIA measurements in health centers. Various bioimpedance instruments are manufactured, the local reference BIA body composition data are available, and a large updated BIA database is ready for international comparisons.

Among major limitations of body composition research in Russia, one can note the lack of validation studies using reference methods, so that foreign regression formulas are used with the double indirect methods, such as anthropometry and BIA, despite the fact that their accuracy has not yet been checked in our population. Conventional reference body composition assessment methods, such as three- or four-component molecular-level models and whole-body in vivo neutron activation analysis, were not applied yet, despite the technical feasibility.

In general, it can be argued that the body composition research in Russia follows the observed global trends. Along with the achievements, there are a number of unresolved methodological and organizational issues. Prospects for further research include validation studies, updating reference population body composition data, and establishing local cut-offs for malnutrition and disease risks. In our view, further development could be facilitated with the establishment of well-equipped Human Body Composition Units in major Russian research centers, such as Moscow State University, which could be assigned a coordinating and methodical role.

## Background

Human body composition studies cover a wide range of fundamental and applied problems of biology and medicine, such as assessment of physical development, diagnosis of obesity and osteoporosis, monitoring of treatment effectiveness, as well as professional and sports selection. The term “body composition” is commonly understood as the structure of body mass. Theoretical and practical aspects of body composition research are presented in numerous publications, a detailed account can be found in [1, 2].

Despite the presence of body composition studies in Russia, there are no current reviews on this topic, and the results are relatively rarely published abroad. Our aim was to describe the history and current state of this research work, to list unresolved issues and to outline possible developmental trends.

## Main text

From the very beginning of the development of Russian anthropology, the study of the geographical diversity of the morphological characteristics of the population in such a vast country as Russia has become extremely important. The study of the geographical distribution of the height of recruits and the factors that affect it was carried out by D.N. Anuchin—the founding father of the Russian anthropological school, the first head of the Department of Anthropology at Moscow State University, and the director of the Research Institute of Anthropology of Moscow State University, which now is named after him. One of his most significant works in this field is the article “On the geographical distribution of height of the male population of Russia (according to the data on universal military service in the Empire for 1874-1883)” [3].

In 1932, a former student and successor of D.N. Anuchin, V.V. Bunak published some important work on the geographical distribution of height in male population of Russia [4]. He analyzed the change in the average height of Russian recruits called up in 1874–1883 and in 1927. He concluded that over the past 50 years they have grown by 2 cm, which he explained by the influence of a genetic factor, namely an increase in heterozygosity due to an increase in marital relations between previously isolated groups of the population due to an increase in its mobility. Bunak published a map of the geographical distribution of height for recruits drafted in 1927 and compared it to what D.N. Anuchin did on data for the period from 1874 to 1883 [4]. The results were very similar, and V.V. Bunak interpreted the results in exactly the same way as D.N. Anuchin: ethno-racial factors have a predominant influence on height variation. V.V. Bunak did not consider the influence of environmental factors in that paper.

After some gap in such studies until the 1960s several important publications appeared dealing with geographical distribution of height and some other body dimensions in different populations of Russia. Among them several volumes of the collected works called “Materials on Physical Development of Children and Adolescents in Some Urban and Rural areas of the USSR”. The first such volume appeared in 1962 under the editorship of A. Goldfeld and others. Several other volumes followed in the successive years [5, 6].

The study of the geographical distribution of anthropometric data in the populations inhabiting different geographical zones was continued. One of the important achievements in this field is connected with the name of the famous Russian anthropologist Tatyana Alexeeva. Together with her students and colleagues, she investigated groups of the rural population, which experienced minimal anthropogenic impact. Based on the ecological rules of Bergmann and Allen, Alexeeva formulated the hypothesis of so-called adaptive types (AT). The AT is not necessarily connected with ethnicity. This is the complex of traits, which independently occurs in similar living conditions in populations that may not be genetically related to each other. The suggested five types were as follows: Arctic AT, Tropical AT, Arid (desert) AT, High-altitudinal AT, and Continental or Moderate AT [7, 8]. Although the concept of ecological rules is important mostly from the point of view of the history of science, its application to the interpretation of specific body morphology in some human populations has been demonstrated in several works. As an example, A.P. Buzhilova and A.V. Kazeeva [9] showed typical characteristics of Arctic AT in modern groups of Chukchi and Eskimo males studied in Eastern Siberia. Bigger weight, smaller height, and an increase of body circumferences were found in the inhabitants of Northern latitudes. Besides pure morphological characteristics, the adaptive traits describing the energy balance of the organism were also analyzed.

The second direction was in the area of applied anthropology for the production of clothing and footwear. Among the works within this framework, an important study of the geographical distribution of anthropological types on the territory of the USSR, which was carried out by A.L. Purundzhan should be mentioned. Under his leadership, staff of the Institute of Anthropology of Lomonosov Moscow State University in 1980–1981 examined 6500 people aged 18 to 20 years. All the material was grouped into 67 ethno-territorial groups, including 31 Russian groups and 15 Ukrainian ones. The corresponding group included those who were born in the territory of the study area and whose parents were of the same nationality. The study program included measurements of 29 traits: 8 on the face and 21 on the body. All measurements were carried out according to the generally accepted anthropometric methodology. Comparing the distribution of body height in the 1870s, 1927, and 1970–1980s, A.L. Purundzhan discovered its stability and continuity. Traditional centers of people with greater and smaller height retained their localization [10, 11]. Currently, the study of the geographical aspects of the growth process is carried out by the Auxology Department in Anuchin Research Institute and Museum of Anthropology. Thus, a study of the spatial variations of growth indicators based on the data from 70 ethno-territorial groups showed the decrease in height from west to east for the indigenous people. At the same time, the west-east gradient is absent in Russian urban children living in the same territories [12]. All of these studies however were only indirectly connected with body composition.

Although studies on body composition became popular in Russia only relatively recently, different schemes and classifications of somatotypes, or as they were called in Russia, “constitutional types,” were thriving from the very beginning of the foundation of Russian anthropology. In a way, these studies could be partly considered as an approach towards the evaluation of body mass components. Indeed, almost in all such classifications, an estimation of fat, muscle, and sometimes bone mass development was included. Historically, a lot of names of those researchers who suggested different typological classifications can be mentioned. Among them are the medical doctors M.V. Chernorutsky, A. Bogomolets, and even the famous physiologist, Nobel prize laureate Ivan Pavlov.

However, in Russian anthropology, the scheme of V.V. Bunak became very popular and widespread [13]. The name of Victor V. Bunak is often mentioned in this review. He is one of the most important figures in Russian anthropology, the founding father of the Russian anthropological school who contributed to many, if not all research fields of biological anthropology, and whose works are in constant use by new generations of scientists.

In general, Bunak’s scheme was similar to that of E. Kretschmer, differing in a more rigid set of constitutional features. Types of constitution in the Bunak’s scheme differed in the degree of development of muscles and fat layer, some additional traits included the shape of the chest, abdomen, and back. Head and face shapes were not taken into account in this scheme, since, in the author’s opinion, they reflected mainly racial rather than constitutional variations. V.V. Bunak identified three main types—thoracic, muscular, and abdominal variants of the physique and four intermediate (thoracic-muscular, muscular-thoracic, muscular-abdominal, and abdominal-muscular) somatotypes. This scheme was designed to define a normal constitution in adult men and was not applicable to women.

Among the constitutional schemes designed to describe the physique of women, the most famous is the typology of I.B. Galant. Based on stature, the degree of fat deposition, and muscle development, as well as the shape of the chest and abdomen, the author identified seven body types, combining them into three groups: leptosomic, mesosomic, and megalosomic types of physique [14]. For children and adolescents in Russian anthropology, the scheme of V.G. Shtefko and A.D. Ostrovsky [15] is widely used, with six main types of normal constitution.

One of the successful domestic schemes for the objective diagnosis of physique types in adults was the classification of V.P. Chtetsov and co-authors, based on 16 measurements characterizing the degree of development of fat, muscle, and bone tissues. This somatotypology used the terminology of the Bunak’s scheme for men and I.B. Galant’s one for women with a slight modification in determining the somatotype in men. For example, the authors divided the thoracic type into thoracic gracile and thoracic broad-boned variants, etc. [16].

One of the most recent and successful approaches to the assessment of somatotype was developed by V.E. Deryabin [17–19]. It can be used for men and women within the age range from 18 to 60 years, as well as for children and adolescents from the age of 1 to 17 years. The scheme is based on variations of measured traits to evaluate the development of skeletal, muscular, and fat body components. The data processing is based on factor analysis. The scheme of V.E. Deryabin demonstrates a wide variety of versions of the physique and provides ample opportunities to study different aspects of its variability.

The most popular scheme of somatotyping developed as the elaboration of W.H. Sheldon’s method by Barbara H. Heath and J.E. Lindsay Carter [20, 21] became popular in Russia only in the late 1980s-1990s, in spite of the fact that Barbara Heath visited Russia several times in the 1960s and 1970s.

Directly connected to body composition methods were the studies of corneal survival by Boris N. Tarusov, who later founded and headed the first University Department of Biophysics in the Soviet Union at the Faculty of Biology of Lomonosov Moscow State University. For this, he suggested using an analog of the bioimpedance phase angle, i.e., the ratio of electrical resistances of the measured tissue at low (10 kHz) and high (1 MHz) frequencies, and in 1939 received the first Soviet patent on bioimpedance [22]. To measure that ratio, the bio-impedance instrument ST-1 was manufactured in the Soviet Union in the 1930s.

In 1941, V.V. Bunak published his seminal book “Anthropometry” [23]. This manual was then very frequently cited in Russia due to suggested standardization of body measurements which is also important for the anthropometric assessment of body composition. The updated local manual on the anthropometry basics was published recently [24], and a reprint of the classical book by V.V. Bunak [23] with modern comments is being prepared.

**Fig. 1:**
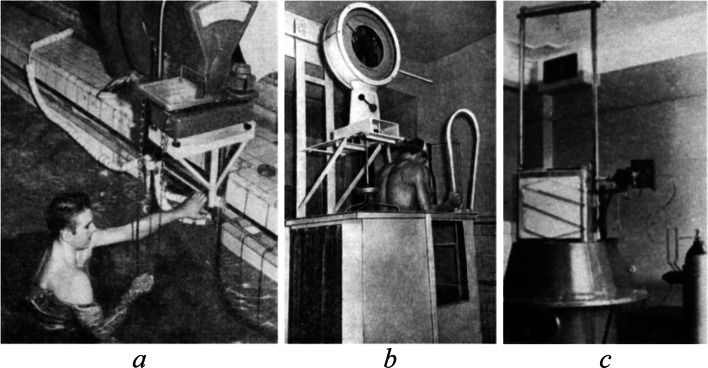
Measurement systems for hydrostatic weighing (**a**, **b**) [27, 28] and volumetry, as modified by A.V. Egorov in 1966 (**c**)

In 1942, simultaneously with the work of A. Behnke [25], P.N. Bashkirov, based on the hydrostatic weighing, studied the factors of variability in body density in 180 adult males [26]. Then and later in the USSR, various measurement systems of hydrostatic weighing and volumetry have been constructed and utilized (Fig. 1).

In 1968, P.N. Bashkirov and others published an important research monograph on body structure and composition in athletes [29]. In 1978, the first domestic application of the whole-body counting of potassium (^40^K) took place [30], and the first (and only) in the USSR D.Sci thesis on body composition (and somatotyping) was defended by V.P. Chtetsov [31]. Later, E.G. Martirosov in his D.Sci thesis studied the peculiarities of body composition and somatotype in athletes depending on the kind of sports as well as sports specialization [32] and published there in the Appendix reference data on body composition of highly qualified athletes specializing in major Olympic sports [33] using the Matiegka [34] four-component tissue-system level model. It can be noted that the Matiegka model prevailed among body composition models in Russia until the late 2000s, and today this method is also not uncommon here (see, e.g., [35, 36]).

Publication of review books [37, 38] also contributed to the general acquaintance of Russian readers with essential aspects and the current state of body composition science and applied research.

**Fig. 2:**
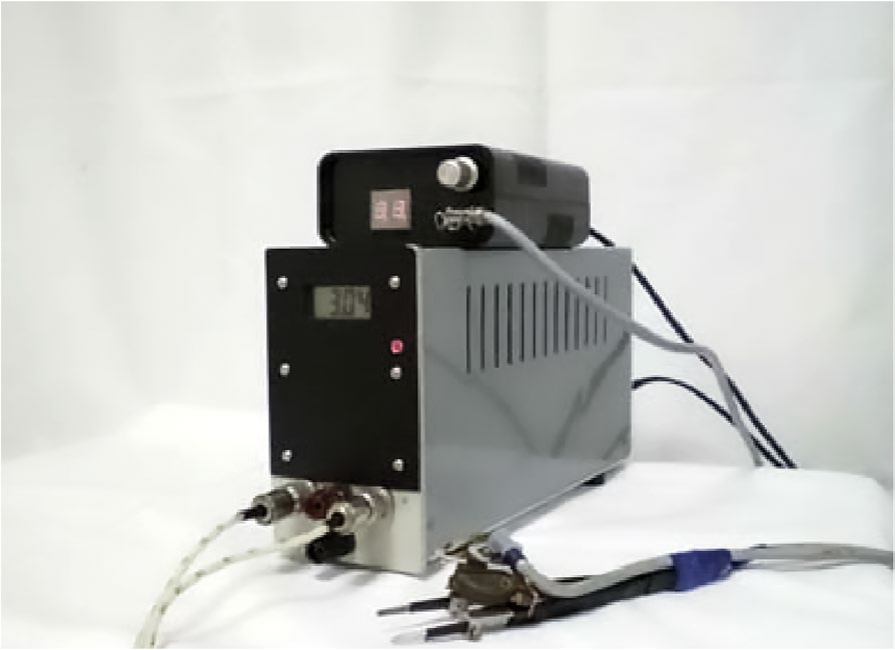
Russian-made experimental bioimpedance meter for surgery and military surgery [39, 40]

A number of experimental and commercial bioimpedance instruments are produced in Russia. The original bioimpedance meter was suggested by the famous Russian anatomist M.R. Sapin and others for use in limb amputations and stomach resections in surgery and military surgery [39, 40] (Fig. 2). Bioimpedance meters for population and clinical studies, as well as sports anthropology and fitness, are manufactured locally by SRC Medas (Moscow) and Diamant LLC (St. Petersburg).

**Fig. 3:**
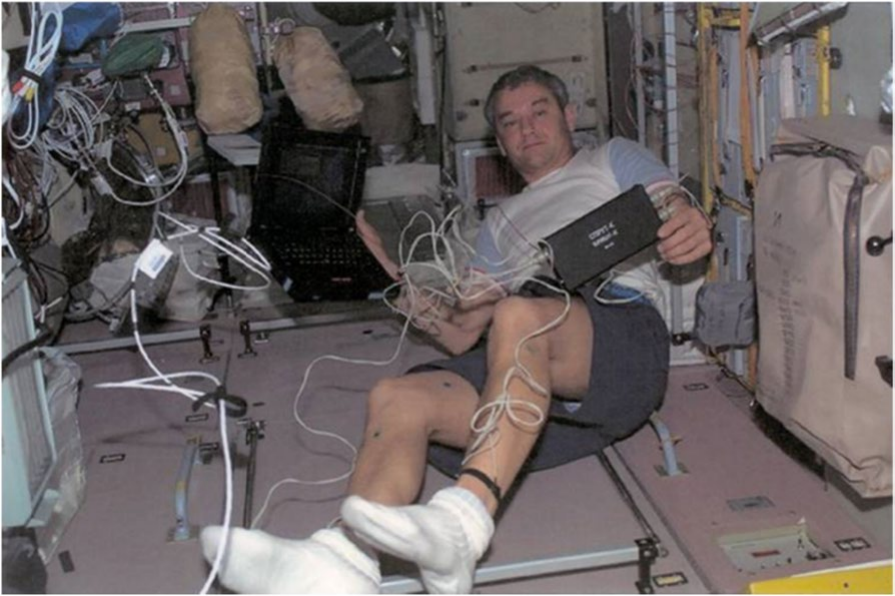
Bioimpedance instrument Octopus-II on the Russian orbital segment of the ISS [38]

The bioimpedance instrument Octopus, a joint product of SRC Medas and the Institute of Biomedical Problems of the Federal Medical-Biological Agency of Russia, has been used since 2002 for the objective control of fluid balance in Russian cosmonauts on board the International Space Station (ISS) (Fig. 3). In microgravity conditions, the centralization of body fluid occurs, so regular physical exercise on special simulators is necessary to restore normal hydration and muscle function.

Ten healthy volunteers who were exposed to 21 days of “dry” immersion simulating the effect of microgravity in Earth conditions showed an increase in the proportion of fat mass as assessed by bioelectrical impedance analysis (BIA) [41]. In the subgroup of individuals with higher relative fat mass and basal metabolic rate, a significant decrease in the proportion of skeletal muscle mass was observed, while in the subgroup with lower relative fat mass and basal metabolic rate the proportion of skeletal muscle mass did not change which was accompanied with an increase in blood insulin [41].

Original equipment and related software were developed and validated for electrical impedance tomography [42–44].

**Fig. 4:**
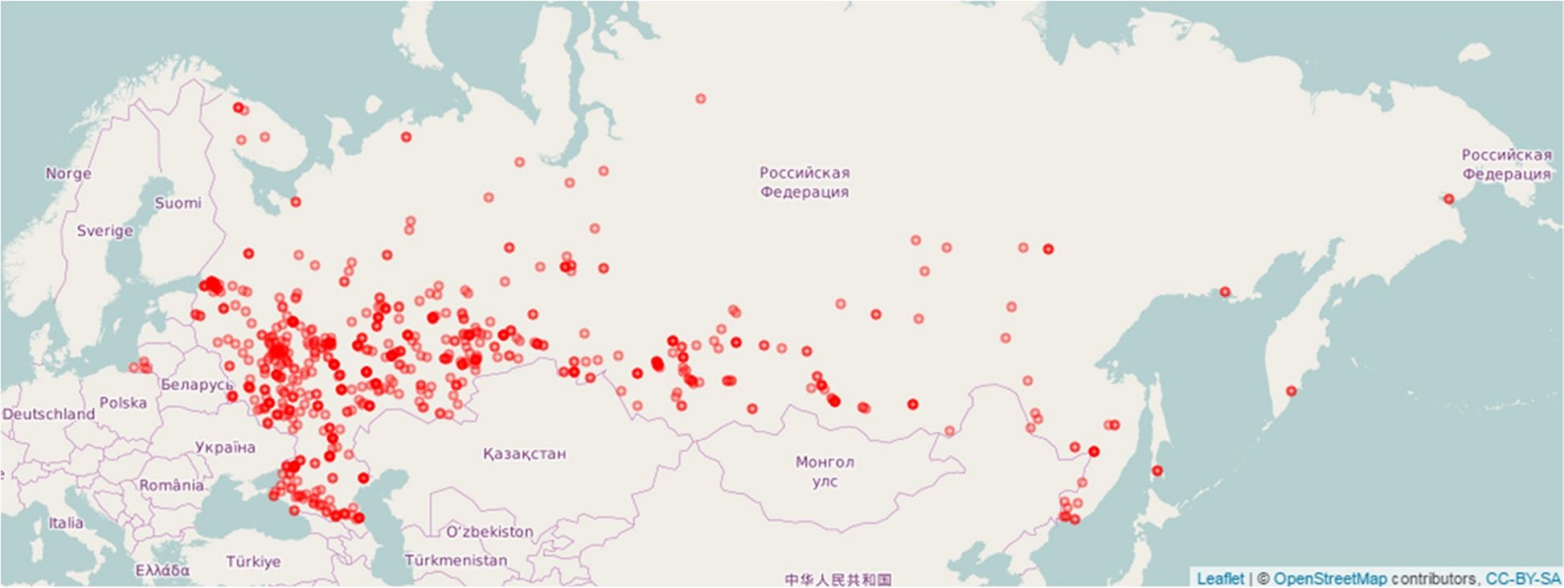
The national network of Health Centers: geographical distribution

A novel stage in body composition research in Russia began with an access to bioimpedance measurement data in Russian health centers (Fig. 4) which were established in 2009–2010 in order to strengthen preventive health care. The feature of that measurement data was a significant proportion of fraud data which led to the development of special software for their preprocessing [45, 46]. The resulting 2010–2012 BIA database contained 809,818 measurement records of individuals aged 5–85 years and was comparable or even exceeded in size of other known BIA databases [47–49]. After the application of the Generalized Additive Models for Location, Scale and Space (GAMLSS) package in R [50], the centile reference tables of sex and age variability of anthropometric parameters and BIA body composition in the Russian population were constructed [51]. Analysis of the data on height, weight, and body mass index in children and adolescents revealed significant differences with the WHO reference data (up to 0.8 standard deviation scores (SDS) in boys and 0.4 SDS in girls) [52] which indicates the relevance of using local references to characterize growth processes. An updated health centers BIA database for 2010–2019 is now formed containing 2,429,977 measurement records.

As was mentioned above, the Heath-Carter anthropometric scheme for the assessment of body physique [20, 21] is now the most commonly used method of somatotyping. This scheme represents the human body type as a three-dimensional vector with the Endomorphy (relative fatness in physique), Mesomorphy (muscular-skeletal robustness), and Ectomorphy (relative linearity of physique) components. Its advantages over other somatotype assessment schemes are the use of continuous and open rating scales and applicability to any ethnic group of both sexes in a wide age range, from 2 to 70+ years. Its limitations include the need to measure 10 specific anthropometric dimensions by a qualified measurer, the dependence on the instruments used, and, hence, relatively low availability for large-scale studies. In our recent publications, simple bioimpedance-based formulae for the assessment of the Endomorphy and Mesomorphy ratings of the Heath-Carter somatotype in ethnically Russian children and adolescents have been constructed and validated [53, 54], and the software for bioimpedance assessment of the somatotype has been developed [55]. These formulae were based on the observed higher linear associations of the Endomorphy and Mesomorphy ratings with the fat- and fat-free mass indices, respectively, as compared to previously tested fat mass and fat-free mass [56–58]. In view of the classical observation on high correlation of the impedance index (i.e. the ratio of height squared to whole-body impedance) with total body volume and, hence, fat-free mass [59], we came to the idea of using the inverse value of the resistance as a predictor of the Mesomorphy and Endomorphy ratings. Initially, the bioimpedance-based equations for the Endomorphy and Mesomorphy ratings (ENDO_BIA_ and MESO_BIA_, respectively) utilized only three measured parameters—namely, subject’s height, weight, and electrical resistance [53]:

$${\displaystyle \begin{array}{l}{\mathrm{ENDO}}_{\mathrm{BIA}}=-\mathrm{3,225}/\mathrm{R}+0.639\times \mathrm{BM}\mathrm{I}-0.416\times \mathrm{BM}-2.20\ \left({r}^2=0.81;\mathrm{SEE}=0.65,\mathrm{n}=2354\right);\\ {}{\mathrm{MESO}}_{\mathrm{BIA}}=\mathrm{2,195}/\mathrm{R}+0.530\times \mathrm{BM}\mathrm{I}-0.097\times \mathrm{BM}-4.55\ \left({r}^2=0.81;\mathrm{SEE}=0.54,\mathrm{n}=2354\right),\end{array}}$$

where R – whole-body electrical resistance (Ohm) measured at 50 kHz according to the conventional tetrapolar wrist-to-ankle electrode configuration, BMI – body mass index (kg/m^2^), BM – body mass (kg), *r*^*2*^ – coefficient of determination, and SEE – standard error of estimate, *n* – sample size. The above formulae for children and adolescents were shown to be accurate in other ethnic groups of Russia, such as Adygeans, Kalmyks, and Tatars [54], and even in various disease states, such as remission of acute lymphoblastic leukemia [60], but were less accurate in students or student athletes [54]. Subsequent addition of the binary variable Sex led to an increase in the accuracy of the Mesomorphy estimate [55]:

$${\displaystyle \begin{array}{l}{\mathrm{ENDO}}_{\mathrm{BIA}}=-\mathrm{2,875}/\mathrm{R}+0.625\times \mathrm{BM}\mathrm{I}-0.042\times \mathrm{BM}-0.23\times \mathrm{Sex}-2.33\ \left({r}^2=0.83;\mathrm{SEE}=0.65,\mathrm{n}=3399\right);\\ {}{\mathrm{MESO}}_{\mathrm{BIA}}=\mathrm{1,467}/\mathrm{R}+0.552\times \mathrm{BM}\mathrm{I}-0.096\times \mathrm{BM}+0.59\times \mathrm{Sex}-4.22\ \left({r}^2=0.86;\mathrm{SEE}=0.47,\mathrm{n}=3399\right),\end{array}}$$

where Sex = 1 (males), 0 (females).

Similar formulae were suggested for adults [61, 62]. As a result, it became possible to utilize regular BIA data (including that of mass population studies) for a reliable assessment of the somatotype and, hence, the Heath-Carter somatotyping has become much more accessible.

**Fig. 5:**
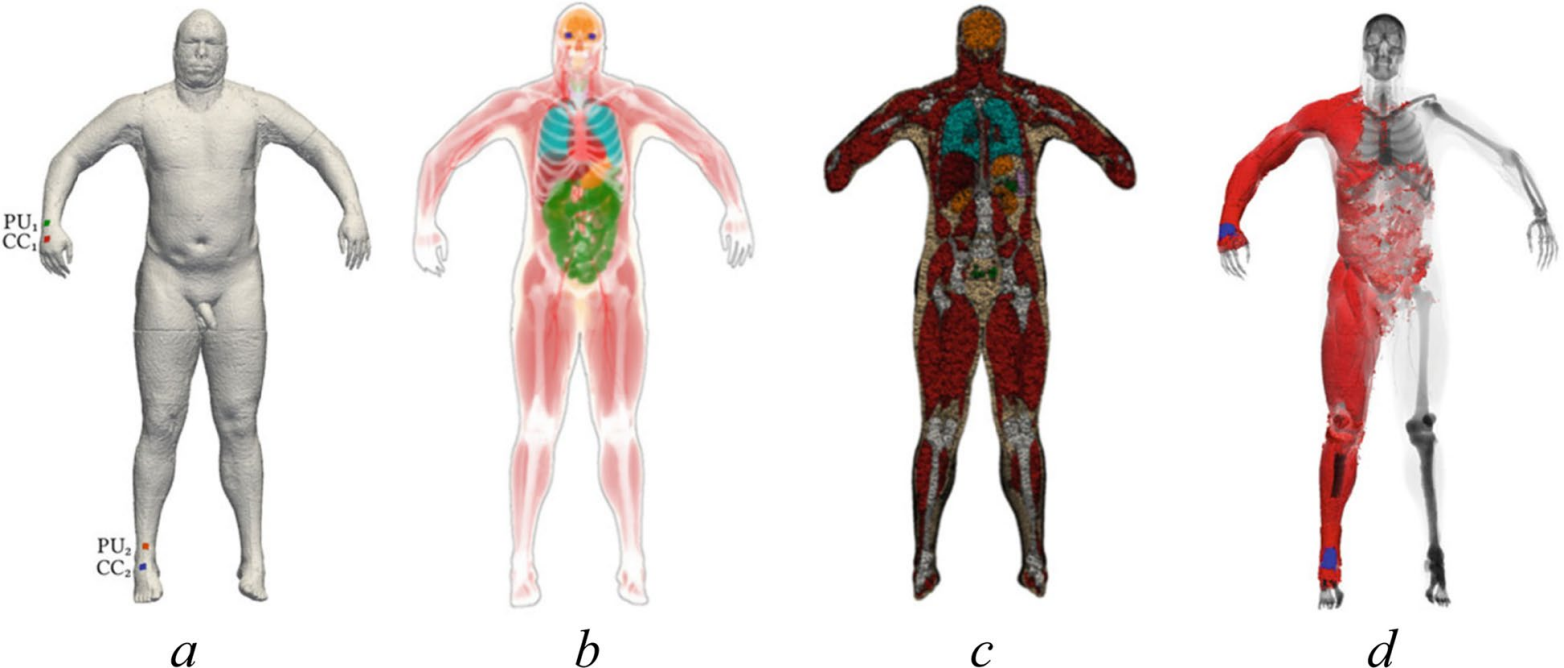
VHP man with stretched arms (**a**), its segmented model (**b**), a cut of the generated mesh by the frontal plane (**c**), and sensitivity field distribution (**d**) for the conventional tetrapolar scheme of bioimpedance measurements [64]

A series of works was carried out at the Institute of Numerical Mathematics of the Russian Academy of Sciences on high-resolution numerical modeling of bioimpedance measurements (see, e.g., [63, 64]). This approach was implemented using anatomically correct 3D model of the human body from the Visible Human Project (VHP) [65] and resulted in the development of FEM-based numerical technology of bioimpedance modeling including 3D image segmentation, adaptive mesh generation, finite element discretization, numerical simulation, and sensitivity analysis thus complementing classical theory [66] and numerical data [67]. An illustration of the workflow of this technology is presented in Fig. 5. In October-November 2021, within the framework of the Russian-Chinese School on mathematical modeling and parallel computing, the first international competition was held among student teams on numerical modeling of bioimpedance measurements [68].

Clinical studies involving bioimpedance body composition assessment in Russia were summarized in [38]. More recent examples include the studies of body composition in childhood cancer [69] and tuberculosis [70]. In contrast to children with cancer, in whom the protein catabolism was usually prevalent at normal BMI values and preserved or even increased fat content, tuberculosis patients showed a decreased BMI in about 30% of the observed cases with the simultaneous reduction in both the lean and fat components. In [71], it was found that low values of the bioimpedance phase angle (below 4°) as measured before the hematopoietic stem cells transplantation were associated with severe complications in early post-transplantation period in all (19 out of 19) cases, while at higher values of phase angle the frequency of severe complications decreased to 50%. Other examples of body composition assessment in clinical studies include the application of double-energy X-ray absorptiometry (DXA) in decompensated liver cirrhosis [72] and rheumatoid arthritis in adults [73], as well as osteogenesis imperfecta in children [74].

With regard to testing new methods, a cross-sectional analysis of the consistency of body composition data provided by the domestic bioimpedance analyzer ABC-02 “Medas” (SRC Medas, Russia) and portable A-mode ultrasound scanner BodyMetrix (IntelaMetrix, USA) was carried out [75]. In frame of the International Atomic Energy Agency coordinated research project E43033 [76], a prospective study of body composition in children and adolescents with acute lymphoblastic leukemia on hematopoietic stem cells transplantation is conducted at the Dmitry Rogachev National Medical Research Center of Pediatric Hematology, Oncology and Immunology in order to test the deuterium dilution method. Also, a cross-sectional study was conducted in 2021 at the Federal Research Institute for Health Organization and Informatics jointly with TechnoAvia company (Moscow branch) and Auxology lab of Moscow State University (MSU) Research Institute and Museum of Anthropology in order to test 3D laser-based photonic scanning technology, as provided by the Anthroscan Vitus XXL instrument (Vitronic, Germany), against the traditional anthropometry.

Single instruments Bod Pod (Life Measurement Instruments, USA) of the air-displacement plethysmography (ADP) method are available in Russia (at least, two devices were purchased in preparation to the 2014 Olympic Games in Sochi, and another one is used at the National Medical Research Center for Rehabilitation and Balneology in elderly), but, most likely, they have not yet been used for research purposes. At the same time, prospective studies of body composition of premature infants were conducted using this method and the Pea Pod (Life Measurement Instruments, USA) device at the National Medical Research Center for Obstetrics, Gynecology and Perinatology named after V.I. Kulakov [77, 78]. It was shown, in particular, that upon reaching full-term age, premature infants had a higher percentage of body fat compared to full-term infants. Also, premature children fed with a specialized mixture showed significantly higher fatness, as well as the levels of insulin, insulin-like growth factor 1, and C-peptide, compared with breastfed children. It was concluded that not only insufficient intrauterine growth, but also the peculiarities of feeding in the neonatal period are important in the development of body composition imbalance and hormonal shifts in premature infants.

The research X-ray densitometer DENIS was developed at the Institute of Nuclear Research of the Russian Academy of Sciences for the assessment of bone tissue density around implants (endoprosthesis) while simultaneously obtaining an image of the examined part of the skeleton and used since 2005 at the National Medical Research Center of Traumatology and Orthopedics named after N.N. Priorov [79]. The authors also reported the availability of an X-ray densitometer DEMON for the diagnosis of bone diseases in mass population studies [80]. Similarly to the densitometer DENIS, this instrument consists of an X-ray emitter, a densitometric wedge, a luminescent gadolinium screen, a short-focus lens and a digital video camera. Clinical trials suggested higher accuracy and stability of the densitometer DEMON compared to the Lunar Prodigy (GE Medical Systems, USA) instrument at a lower (2.5 times) cost. This was achieved due to the absence of scanning, and, consequently, cheaper mechanics, reduced requirements for the stability of the X-ray tube, and no necessity for complex calibrations.

## Discussion

It can be seen that anthropometry and bioimpedance analysis are mainly used to assess body composition in Russia. Other methods, such as DXA, are utilized less often, mainly in clinical studies. According to the established tradition, the Matiegka four-component tissue-system level model is still quite widely used locally. It can be noted that this model still provides reasonable indexes of adiposity and muscularity despite the incompatibility with the molecular-level body composition models and unclear accuracy.

From our point of view, a serious lag in body composition research in Russia occurred as a result of a missed technological leap in this field in the mid and late 1980s, during the decline of the USSR. Among major limitations of body composition research in Russia, one can note the lack of validation studies using reference methods, so that foreign regression formulas are used with the double indirect methods, such as anthropometry and BIA, despite the fact that their accuracy has not yet been studied in our population. Conventional reference body composition methods, such as three- or four-component molecular-level models and whole-body in vivo neutron activation analysis, were not applied yet, despite the technical feasibility, and new reference methods are not being developed. In our view, this is one of the main reasons for the relatively low availability of domestic research internationally, where application of reference body composition methods is often necessary.

At the same time, the achievements include common use of comprehensive anthropometry in anthropological studies, some advancements in clinical studies, approbation of potentially important methods such as the deuterium dilution method and three-dimensional laser-based photonic scanning, and ongoing mass population BIA measurements in health centers. Various bioimpedance instruments are manufactured, the local reference BIA body composition data are available, and a large updated BIA database is ready for international comparisons.

## Conclusions

In general, it can be argued that the body composition research in Russia follows the observed global trends. Along with the achievements, there are a number of unresolved methodological and organizational issues. Prospects for further research include validation studies, updating reference population body composition data, and establishing local cut-offs for malnutrition and disease risks. In our view, further development could be facilitated with the establishment of well-equipped Human Body Composition Units in major Russian research centers, such as MSU, which could be assigned a coordinating and methodical role.

This manuscript presents our subjective and inevitably incomplete view at a glance on the state of body composition studies in Russia. We invite other authors to share their thoughts and criticism.

## Data Availability

Not applicable.

## Abbreviations

ADP: Air-displacement plethysmography
AT: Adaptive types
BIA: Bioelectrical impedance analysis
BM: Body mass
BMI: Body mass index
DXA: Double-energy X-ray absorptiometry
FEM: Finite element method
GAMLSS: Generalized Additive Models for Location, Scale and Space
ISS: International Space Station
MSU: Moscow State University
*n*: Sample size
*R*: Whole-body electrical resistance
*r*^2^: coefficient of determination
SDS: Standard deviation score
SEE: Standard error of estimate
VHP: Visible Human Project
WHO: World Health Organization
